# Nanomaterials in Liquid Crystals

**DOI:** 10.3390/nano8070453

**Published:** 2018-06-21

**Authors:** Ingo Dierking

**Affiliations:** School of Physics and Astronomy, University of Manchester, Oxford Road, Manchester M13 9PL, UK; ingo.dierking@manchester.ac.uk

Liquid crystals are often identified with the development of the flat panel television and computer screens that we all use on a daily basis. Despite their enormous success in this area, liquid crystal research is by far not exhausted and has reinvented itself, spearheading into other fields of research, due to their properties of self-organization, their fascinating optic and electro-optic properties, and their easy deformability and reorientation via electric, magnetic, mechanical and other external fields. Novel effects are being discovered, new modern and self-organized materials are constantly being developed and a whole range of non-display applications is being proposed, which are on the borderline between nanotechnology and soft condensed matter. Liquid crystals are also being employed as a vehicle to study fundamental physical questions, and proceeding into the areas of biology, nature and life. In this Special Issue of *Nanomaterials*, illustrative examples are introduced, which draw on aspects of self-organization of liquid crystals, colloidal ordering of nanoparticles, and the formation of anisotropic, liquid crystalline phases from nanoparticles. An exhaustive treatment of these topics up to about 2015 can be found in the two volume handbook edited by Lagerwall and Scalia [1].

Liquid crystals [2,3,4] are partially ordered, anisotropic fluids, which are thermodynamically located between the three dimensional solid crystal and the flow governed liquid. They exhibit orientational or low dimensional positional order of their long molecular axis or the molecular centres of mass, respectively, which results in anisotropic physical properties, such as refractive index, viscosity, elastic constant, electric conductivity, or magnetic susceptibility, while retaining the ability to flow. Liquid crystals are part of the ever growing and increasingly important family of soft condensed matter materials [5,6,7,8,9]. Two general classes of liquid crystals are mostly distinguished, *thermotropic* materials [10,11], which exhibit the liquid crystalline state exclusively on temperature variation, and *lyotropic* liquid crystals [12,13], where the formation of liquid crystal phases is achieved by concentration variation of shape anisotropic dopant materials in an isotropic carrier or host fluid. The latter type is most often composed from amphiphilic molecules in water, but can also be observed by dispersing anisotropic colloidal particles in an isotropic liquid [14]. A classic example is that of vanadium pentoxide, V_2_O_5_, which had already been shown about a century ago by Freundlich [15] to be anisotropic. Nevertheless, also many other minerals and clays, lead to inorganic liquid crystals, as has been reviewed by Sonin [16]. 

Thermotropic dispersions and lyotropic liquid crystalline behaviour have also been reported for carbon based materials, for example involving single-walled and multi-walled nanotubes [17,18,19,20,21,22] for electrically and magnetically addressable molecular switches. Also lyotropic graphene oxide [23,24,25,26,27,28] has been explored in a variety of host liquids for possible electro-optic applications based on the Kerr effect. Further reports discuss inorganic nanorods [29,30,31,32,33], ferroelectric particles [34,35,36] and magnetic nanorods [37,38] and platelets for ferromagnetic nematics. Also the incorporation of gold nanoparticles [39,40,41] into liquid crystals or indeed mesogenic molecules has become popular, especially for applications in plasmonics. Furthermore, carbon materials such as fullerenes [42,43] are also incorporated into liquid crystal forming molecules. The general reasons for dispersing colloids in liquid crystals by a variety of different methods and procedures [44,45], are to tune the liquid crystal properties, to add functionality, or to exploit the self-organization of the liquid crystal and use the order of the host as a template to transfer order onto dispersed nanomaterials.

The mechanical properties, dominated by extremely small elastic constants when compared to solid state materials, are another of the characteristics of all liquid crystals. The fact that elastic constants are very small, implies that topological defects in liquid crystals extend over large, macroscopic distances, so that they can easily be observed in polarizing microscopy. This in turn leads to textures with topological defects of strength s = ±1/2 (two-fold brushes) and s = ±1 (four-fold brushes), where defects of opposite sign and equal strength attract each other and annihilate [46,47,48]. Defect annihilation in liquid crystals is a means to study fundamental dynamical theories, collectively known as the Kibble-Zurek mechanism [49,50] in an elegant way. From a more practical point of view, such defect structures can be stabilized by confinement in one-, or two-dimensional arrays for optical elements [51] or to act as biological surface sensors [52].

In the theoretical work of Holger Stark [53] and the experiments of Igor Musevic et al. [54] it was shown that defects are also induced when micro-spheres are placed in a well oriented nematic liquid crystal. Different types of defects can be observed for different anchoring conditions on the micro-particles, called hedgehog and Saturn ring defects. Further, the attractive bipolar or quadrupolar force between defects can also lead to the phenomenon of chaining, forming linear chains of colloids and zigzag-shaped chains, respectively. This can also be observed for rod-shaped colloidal particles [55,56]. Even two-dimensional arrays of nanomaterials can be formed, as was also confirmed elegantly through the computer simulation of the group around Slobodan Zumer [57]. The field of colloidal interactions studied in liquid crystals was fuelled by the initial observations in the pioneering work of Poulin et al. [58], who investigated nematic-water emulsions with water droplets acting as colloidal particles and determining the force that attracts two droplet colloids [59].

It is thus clear that the fields of nanomaterials dispersed in liquid crystals, as well as that of the formation of lyotropic liquid crystal phases by dispersing anisotropic nanomaterials in isotropic host liquids will continue to grow and attract interest from a wider community. This will include the synthesis of nanoparticle containing mesogens as much as the development of novel methods of dispersion of nanomaterials in liquid crystal hosts, both thermotropic and lyotropic. Different materials will be used, carbon based nanomaterials in zero-, one-, and two dimensions, minerals and clays, and synthetic nanorods, as well as biological nanoparticles. Different functionalities will be explored, ferroelectricity, ferromagnetism, semiconductivity, chirality, quantum dots, or plasmonic properties. And experiments will be joined by theory and computer simulations to eventually produce applications which will exploit the best of all areas, liquid crystals and nanomaterials, not only to improve display applications, but also to generate novel applications in the fields of optics, sensors, medicine and related fields. 

I would like to thank all authors of this Special Issue of *Nanomaterials* for their contributions and all referees for their valuable comments and suggestions, as well as the editorial office for their constant and swift support.

## References

[1] Lagerwall J.P.F., Scalia G. (2016). Liquid Crystals with Nano and Micro-Particles.

[2] Collings P.J., Hird M. (1997). Introduction to Liquid Crystals: Chemistry and Physics.

[3] Chandrasekhar S. (1992). Liquid Crystals.

[4] Singh S. (2002). Liquid Crystals: Fundamentals.

[5] Jones R.A.L. (2002). Soft Condensed Matter.

[6] Hamley E.W. (2007). Introduction to Soft Matter: Revised Edition.

[7] Kleman M., Lavrentovich O.D. (2003). Soft Matter Physics: An Introduction.

[8] Hirst L.S. (2013). Fundamentals of Soft Matter Science.

[9] Terentjev E.M., Weitz D.A. (2015). The Oxford Handbook of Soft Condensed Matter.

[10] De Gennes P.G., Prost J. (1993). The Physics of Liquid Crystals.

[11] Dierking I. (2003). Textures of Liquid Crystals.

[12] Petrov G. (1999). The Lyotropic State of Matter.

[13] Neto A.M.F., Salinas S.R.A. (2005). The Physics of Lyotropic Liquid Crystals.

[14] Dierking I., Al-Zangana S. (2017). Lyotropic Liquid Crystal Phases from Anisotropic Nanomaterials. Nanomaterials.

[15] Diesselhorst H., Freundlich H. (1915). On the double refraction of vanadine pentoxydsol. Phys. Z..

[16] Sonin A.S. (1998). Inorganic lyotropic liquid crystals. J. Mater. Chem..

[17] Dierking I., Scalia G., Morales P., LeClere D. (2004). Aligning and reorienting carbon nanotubes with nematic liquid crystals. Adv. Mater..

[18] Dierking I., Scalia G., Morales P. (2005). Liquid crystal–carbon nanotube dispersions. J. Appl. Phys..

[19] Lagerwall J., Scalia G., Haluska M., Dettlaf-Weglikowska U., Roth S., Giesselmann F. (2007). Nanotube alignment using lyotropic liquid crystals. Adv. Mater..

[20] Kumar S., Bisoyi H.K. (2007). Aligned carbon nanotubes in the supramolecular order of discotic liquid crystals. Angew. Chem. Int. Ed..

[21] Song W., Kinloch I.A., Windle A.H. (2003). Nematic liquid crystallinity of multiwall carbon nanotubes. Science.

[22] Badaire S., Zakri C., Maugey M., Derre A., Barisci J.N., Wallace G., Poulin O. (2005). Liquid crystals of DNA-stabilized carbon nanotubes. Adv. Mater..

[23] Kim J.E., Han T.H., Lee S.H., Kim J.Y., Ahn C.W., Yun J.M., Kim S.O. (2011). Graphene oxide liquid crystals. Angew. Chem. Int. Ed..

[24] Xu Z., Gao C. (2011). Aqueous liquid crystals of graphene oxide. ACS Nano.

[25] Shen T.Z., Hong S.H., Song J.K. (2014). Electro-optical switching of graphene oxide liquid crystals with an extremely large Kerr coefficient. Nat. Mater..

[26] Al-Zangana S., Iliut M., Turner M., Vijayaraghavan A., Dierking I. (2016). Properties of a thermotropic nematic liquid crystal doped with graphene oxide. Adv. Opt. Mater..

[27] Al-Zangana S., Iliut M., Turner M., Vijayaraghavan A., Dierking I. (2017). Confinement effects on lyotropic nematic liquid crystal phases of graphene oxide dispersions. 2D Mater..

[28] Narayan R., Kim J.E., Kim J.Y., Lee K.E., Kim S.O. (2016). Liquid Crystals: Graphene Oxide Liquid Crystals: Discovery, Evolution and Applications (Adv. Mater. 16/2016). Adv. Mater..

[29] Saliba S., Mingotaud C., Kahn M.L., Marty J.-D. (2013). Liquid crystalline thermotropic and lyotropic nanohybrids. Nanoscale.

[30] Zhang S., Majewski P.W., Keskar G., Pfefferle L.D., Osuji C.O. (2011). Lyotropic self-assembly of high-aspect-ratio semiconductor nanowires of single-crystal ZnO. Langmuir.

[31] Ren Z., Chen C., Hu R., Mai K., Qian G., Wang Z. (2012). Two-Step Self-Assembly and Lyotropic Liquid Crystal Behavior of TiO_2_ Nanorods. J. Nanomater..

[32] Li L.-S., Walda J., Manna L., Alivisatos A.P. (2002). Semiconductor nanorod liquid crystals. Nano Lett..

[33] Thorkelsson K., Bai P., Xu T. (2015). Self-assembly and applications of anisotropic nanomaterials: A review. Nano Today.

[34] Li F.H., West J., Glushchenko A., Cheon C.I., Reznikov Y. (2006). Ferroelectric nanoparticle/liquid-crystal colloids for display applications. J. Soc. Inf. Disp..

[35] Basu R. (2014). Soft memory in a ferroelectric nanoparticle-doped liquid crystal. Phys. Rev. E.

[36] Al-Zangana S., Turner M., Dierking I. (2017). A comparison between size dependent paraelectric and ferroelectric BaTiO_3_ nanoparticle doped nematic and ferroelectric liquid crystals. J. Appl. Phys..

[37] Podoliak N., Buchnev O., Bavykin D.V., Kulak A.N., Kaczmarek M., Sluckin T.J. (2012). Magnetite nanorod thermotropic liquid crystal colloids: Synthesis, optics and theory. J. Colloid Interface Sci..

[38] Mertelj A., Lisjak D., Drofenik M., Copic M. (2013). Ferromagnetism in suspensions of magnetic platelets in liquid crystal. Nature.

[39] Cseh L., Mehl G.H. (2006). The design and investigation of room temperature thermotropic nematic gold nanoparticles. J. Am. Chem. Soc..

[40] Liu Q.K., Cui Y.X., Gardner D., Li X., He S.L., Smalyukh I.I. (2010). Self-Alignment of Plasmonic Gold Nanorods in Reconfigurable Anisotropic Fluids for Tunable Bulk Metamaterial Applications. Nano Lett..

[41] Dintinger J., Tang B.J., Zeng X.B., Liu F., Kienzler T., Mehl G.H., Ungar G., Rockstuhl C., Scharf T. (2013). A Self-Organized Anisotropic Liquid-Crystal Plasmonic Metamaterial. Adv. Mater..

[42] Sawamura M., Kawai K., Matsuo Y., Kanie K., Kato T., Nakamura E. (2002). Stacking of conical molecules with a fullerene apex into polar columns in crystals and liquid crystals. Nature.

[43] Lehmann M., Huegel M. (2015). A Perfect Match: Fullerene Guests in Star-Shaped Oligophenylenevinylene Mesogens. Angew. Chem. Int. Ed..

[44] Hegmann T., Qi H., Marx V.M. (2007). Nanoparticles in liquid crystals: Synthesis, self-assembly, defect formation and potential applications. J. Inorg. Organomet. Polym. Mater..

[45] Stamatoiu O., Mirzaei J., Feng X., Hegmann T. (2012). Nanoparticles in liquid crystals and liquid crystalline nanoparticles. Top. Curr. Chem..

[46] Chuang I., Durrer R., Turok N., Yurke B. (1991). Cosmology in the laboratory: Defect dynamics in liquid crystals. Science.

[47] Dierking I., Marshall O., Wright J., Bulleid N. (2005). Annihilation dynamics of umbilical defects in nematic liquid crystals under applied electric fields. Phys. Rev. E.

[48] Dierking I., Ravnik M., Lark E., Healey J., Alexander G.P., Yeomans J.M. (2012). Anisotropy in the annihilation dynamics of umbilic defects in nematic liquid crystals. Phys. Rev. E.

[49] Kibble T.W.B. (1976). Topology of cosmic domains and strings. J. Phys. A.

[50] Zurek W.H. (1985). Cosmological experiments in superfluid helium. Nature.

[51] Migara L.K., Lee C.-M., Kwak K., Lee H., Song J.-K. (2018). Tunable optical vortex arrays using spontaneous periodic pattern formation in nematic liquid crystal cells. Curr. Appl. Phys..

[52] Brake J.M., Daschner M.K., Luk Y.Y., Abbott N.L. (2003). Biomolecular interactions at phospholipid-decorated surfaces of liquid crystals. Science.

[53] Stark H. (2001). Physics of colloidal dispersions in nematic liquid crystals. Phys. Rep..

[54] Musevic I. (2017). Liquid Crystal Colloids.

[55] Tkalec U., Skarabot M., Musevic I. (2008). Interactions of micro-rods in a thin layer of a nematic liquid crystal. Soft Matter.

[56] Dierking I., Heberle M., Osipov M.A., Giesselmann F. (2017). Ordering of ferromagnetic nanoparticles in nematic liquid crystals. Soft Matter.

[57] Ravnik M., Skarabot M., Zumer S., Tkalec U., Poberaj I., Babic D., Osterman N., Musevic I. (2007). Entangled nematic colloidal dimers and wires. Phys. Rev. Lett..

[58] Poulin P., Stark H., Lubensky T.C., Weitz D.A. (1997). Novel colloidal interactions in anisotropic fluids. Science.

[59] Poulin P., Cabuil V., Weitz D.A. (1997). Direct measurement of colloidal forces in an anisotropic solvent. Phys. Rev. Lett..

